# Ethical and policy issues in cluster randomized trials: rationale and design of a mixed methods research study

**DOI:** 10.1186/1745-6215-10-61

**Published:** 2009-07-28

**Authors:** Monica Taljaard, Charles Weijer, Jeremy M Grimshaw, Judith Belle Brown, Ariella Binik, Robert Boruch, Jamie C Brehaut, Shazia H Chaudhry, Martin P Eccles, Andrew McRae, Raphael Saginur, Merrick Zwarenstein, Allan Donner

**Affiliations:** 1 Ottawa Hospital Research Institute, Clinical Epidemiology Program, Ottawa Hospital, 1053 Carling Avenue, Civic Campus, C409, Ottawa, ON K1Y 4E9, Canada; 2 Department of Epidemiology and Community Medicine, University of Ottawa, Ottawa, Canada; 3 Departments of Philosophy and Medicine, Joseph L. Rotman Institute of Science and Values, University of Western Ontario, London, Ontario, N6A 3K7, Canada; 4 Ottawa Hospital Research Institute, Clinical Epidemiology Program, 1053 Carling Avenue, Civic Campus, ASB 2-018, Ottawa, Ontario K1Y 4E9, Canada; 5 Department of Medicine, Faculty of Medicine, University of Ottawa, Ottawa, Canada; 6 Center for Studies in Family Medicine, Department of Family Medicine, Schulich School of Medicine and Dentistry, 245-100 Collip Circle, London, Ontario, N6G 4X8, Canada; 7 Joseph L. Rotman Institute of Science and Values, Department of Philosophy, University of Western Ontario, London, Ontario, N6A 3K7, Canada; 8 Graduate School of Education and Statistics Department, Wharton School, University of Pennsylvania, 3700 Walnut Street; Philadelphia; Pennsylvania 19104, USA; 9 Ottawa Hospital Research Institute, Clinical Epidemiology Program, Ottawa Hospital, 1053 Carling Avenue, ASB 2-004, Ottawa, Ontario K1Y 4E9, Canada; 10 Department of Epidemiology and Community Medicine, University of Ottawa, Ottawa, Canada; 11 Ottawa Hospital Research Institute, Clinical Epidemiology Program; Ottawa Hospital, 1053 Carling Avenue, F663a; Ottawa, ON K1Y 4E9, Canada; 12 Department of Epidemiology and Community Medicine, University of Ottawa, Ottawa, Canada; 13 Institute of Health & Society, Newcastle University, 21 Claremont Place, Newcastle upon Tyne, NE2 4AA, UK; 14 Joseph L. Rotman Institute of Science and Values, Department of Epidemiology and Biostatistics, Ontario, N6A 3K7, Canada; 15 Department of Emergency Medicine, London Health Sciences Centre, 800 Commissioners Rd East, London, ON, N6A 5W9, Canada; 16 Department of Medicine, University of Ottawa and Ottawa Hospital; Ottawa Hospital Research Institute, 1053 Carling Avenue, Ottawa, ON K1Y 4E9, Canada; 17 Centre for Health Services Sciences, Sunnybrook Health Sciences Centre, 2075 Bayview Avenue, Toronto, M4N 3M5, Canada; 18 Department of Epidemiology and Biostatistics, University of Western Ontario, Kresge Building, Room K201, London, Ontario, N6A 5C1, Canada; 19 Robarts Clinical Trials, Robarts Research Institute, London, ON, N6A 5K8, Canada

## Abstract

**Background:**

Cluster randomized trials are an increasingly important methodological tool in health research. In cluster randomized trials, intact social units or groups of individuals, such as medical practices, schools, or entire communities – rather than individual themselves – are randomly allocated to intervention or control conditions, while outcomes are then observed on individual cluster members. The substantial methodological differences between cluster randomized trials and conventional randomized trials pose serious challenges to the current conceptual framework for research ethics. The ethical implications of randomizing groups rather than individuals are not addressed in current research ethics guidelines, nor have they even been thoroughly explored. The main objectives of this research are to: (1) identify ethical issues arising in cluster trials and learn how they are currently being addressed; (2) understand how ethics reviews of cluster trials are carried out in different countries (Canada, the USA and the UK); (3) elicit the views and experiences of trial participants and cluster representatives; (4) develop well-grounded guidelines for the ethical conduct and review of cluster trials by conducting an extensive ethical analysis and organizing a consensus process; (5) disseminate the guidelines to researchers, research ethics boards (REBs), journal editors, and research funders.

**Methods:**

We will use a mixed-methods (qualitative and quantitative) approach incorporating both empirical and conceptual work. Empirical work will include a systematic review of a random sample of published trials, a survey and in-depth interviews with trialists, a survey of REBs, and in-depth interviews and focus group discussions with trial participants and gatekeepers. The empirical work will inform the concurrent ethical analysis which will lead to a guidance document laying out principles, policy options, and rationale for proposed guidelines. An Expert Panel of researchers, ethicists, health lawyers, consumer advocates, REB members, and representatives from low-middle income countries will be appointed. A consensus conference will be convened and draft guidelines will be generated by the Panel; an e-consultation phase will then be launched to invite comments from the broader community of researchers, policy-makers, and the public before a final set of guidelines is generated by the Panel and widely disseminated by the research team.

## Background

### Cluster randomized trials

In recent years, there has been a growing interest in generating dependable evidence about the effectiveness of health policies, programs and practices, using randomized designs. In some studies, randomization at the individual (patient) level may not be feasible because the intervention is designed to be implemented at the group level or because the hypothesized mechanism of action of the intervention operates at the group level. In health services implementation research [1] for example, the intervention may be administered to the health professional or may involve changes to the health care organization; and in trials of infectious disease interventions, a vaccine may be administered at the individual level but its effects observed among those in the wider community as a consequence of herd immunity. Randomization at the individual level may also be undesirable for methodological reasons such as the need to avoid contamination (for example, in trials of behavioural interventions) when individuals in close proximity are randomized to competing interventions. Finally, randomizing individuals may complicate the trial organization and implementation, for example, in developing nation settings where special equipment or personnel are required or permission from political authorities must be obtained to conduct the trial. The cluster randomized design [2] has thus become an increasingly important methodological tool in health and health services research: in cluster randomized trials (also known as group randomized or place randomized trials), intact social units or clusters of individuals, such as medical practices, communities, schools or villages are randomized to intervention or control conditions. The interventions may be delivered to the entire randomized group as a unit, or to individuals within each group, but all members of a group receive the same intervention; outcomes are then observed on individual cluster members (or subsamples of members) to evaluate the effect of the experimental intervention. Note that, although outcomes are observed on individuals, they may be aggregated at the cluster-level, for example, percentage of X-ray requests by physicians.

### Ethical challenges in cluster randomized trials

The substantial methodological differences between cluster randomized trials and conventional randomized trials pose serious challenges to the current conceptual framework for research ethics. Contemporary research ethics is largely structured around the protection of the autonomy and welfare interests of individual research subjects. Ethical principles governing the conduct of clinical research are laid out in the Belmont Report [3]. The ethical principle of respect for persons means that choices of autonomous individuals ought to be taken seriously and that persons who cannot responsibly choose for themselves are entitled to protection. This principle is the source of the moral rules requiring informed consent from research subjects and protection of confidential health information. The ethical principle of beneficence means that researchers have an obligation to protect subjects from avoidable harm and, where possible, to promote their welfare interests. It is the source of a variety of moral rules that guide the ethical analysis of study benefits and harms [4]. The ethical principle of justice means that study subjects ought to be treated fairly. It grounds requirements that the vulnerable, such as children or incapable adults, not be included as a population of mere convenience. Recently, a novel ethical principle of respect for communities has been proposed [5]. It requires that investigators have an obligation to respect communal values, protect and empower social institutions, and, where applicable, abide by the decisions of legitimate communal authorities.

The ethical implications of randomizing groups rather than individuals have as yet not been thoroughly explored [6-11]. This is illustrated by the ethical issues arising from three different cluster randomized trials: In the Community Intervention Trial for Smoking Cessation (COMMIT) [12], twenty-two cities in the USA and Canada were randomized to either a community-level antismoking intervention delivered through mass media, health care professionals and worksites, or to a no-intervention control. Baseline data were collected from a random sample of citizens using a telephone survey, and a sample of smokers identified and followed with annual telephone surveys to ascertain smoking cessation status. The investigators interacted with the community by forming a board of community representatives. Individual respondents were not aware that they were involved in a trial, although they provided verbal consent to complete the telephone surveys. Questions raised by this study include: Is consent required from the communities involved? [8] If so, from whom? Is the municipal government empowered to make these decisions? [13] What criteria ought community decision-makers use? [10] When the intervention is targeted at the entire community, it may be impossible to obtain individual informed consent from all citizens – is this ethically legitimate, or does an inability to consent to or opt out of a community-level intervention violate the rights of individual citizens? [13]

Analogous questions arise in quality improvement interventions where the intervention may be targeted at health professionals but affect patient care. For example, in a cluster randomized trial to improve adherence to guidelines for hypertension drug prescribing, physicians and nurse practitioners were randomized to receive either general guideline education or education plus patient-specific reminders about hypertension [14]. Outcomes included overall compliance with guidelines and adequacy of patients' blood pressure control as ascertained by review of patients' medical records. The requirement for informed consent for both practitioners and individual subjects was waived by the Research Ethics Board (REB). Questions raised by this study include: Who are the research subjects: health professionals, patients, or both? Should consent be required from the participating health professionals [9] or is there a professional obligation for health professionals to participate in quality improvement research that obviates the need for their informed consent? If the study is using routinely-collected data with identifying information removed, is patient consent required? How are we to understand this study with respect to the analysis of benefits and harms? [13]

A third, very different study randomized villages in Nepal to provide nutritional supplements to women of child-bearing age [15]. Villages were randomized to one of four study arms: vitamin A supplements, β-carotene supplements, both supplements, or placebo. The outcome of interest was mortality associated with pregnancy and childbirth. Community leaders agreed to randomization of communities, while individual women gave verbal consent to receive the supplements and provide data. A sample of women who became pregnant underwent further investigations, including blood sampling. Mortality and other variables were collected prospectively by study workers. Further information regarding fatalities was obtained from interviews with the families of any subjects who died. While this study shares many of the issues raised in the first example, additional questions include: Do investigators bear any special obligations to subjects because of the developing nation setting [5]? Is there an obligation to offer ancillary benefits to the control arm or to all study arms (e.g., additional health care unrelated to the study question) [11]?

### Inadequacy of Current Research Ethics Guidelines

Given the uncertainty in the literature as to how to address the ethical problems presented by cluster randomized trials, it is not surprising that current research ethics guidelines do not address these issues. Relevant international and national guidelines include the World Medical Association *Declaration of Helsinki *[16], the Council of International Organizations of Medical Sciences (CIOMS) *International Ethical Guidelines for Biomedical Research Involving Human Subjects *[17], the International Conference of Harmonization *Guideline for Good Clinical Practice *[18], CIOMS 1991 *International Guidelines for Ethical Review of Epidemiological Studies *[19], Canadian *Tri-Council Policy Statement *[20], United Kingdom *Medical Research Council Guidelines for Good Clinical Practice in Clinical Trials *[21], and US federal regulations [22]. These guiding documents were designed to protect the welfare and liberty interests of individual research subjects, but little guidance is provided with respect to community-based research, let alone cluster randomized trials. The sole exception is the UK Medical Research Council document (*Cluster Randomized Trials: Methodological and Ethical Considerations*) [8]; however, this document does not address the broad scope of ethical issues identified above, and its applicability to the regulatory environments of other countries is uncertain.

Researchers need direction on these ethical challenges in order to guide the ethically appropriate design and conduct of cluster randomized trials. REBs may be unfamiliar with this increasingly important study methodology. In the absence of formal guidelines for cluster randomized trials, REBs may fail to consider all of the relevant ethical issues generated by a study protocol, resulting in inadequate subject protection. Variable interpretation of ethical requirements for cluster randomized trials may lead to problems initiating multi-jurisdictional cluster randomized trials, and to unequal treatment of subjects in different jurisdictions. For example, Chaney et al. [23] reported on a series of health services implementation research studies involving primary care centres in the USA in which a combined total of more than 100 ethics review applications, amendments, and renewals were submitted at 17 research sites. Substantial variation in process and outcomes among sites was reported, with 35% of sites considering the research as exempt from review, 41% granting expedited review, and 18% requiring full review; approximately half of the REBs had ethical concerns necessitating changes to consent documents, and the number of days from submission to approval varied widely among sites, ranging from 3 to 82 days. Dziak et al. [24] reported on the ethics review process in a health services research study involving 15 primary care sites; REBs varied in the types of review required, and the number of days from submission to approval ranged from 5 to 172 days. According to the authors, variability in requirements for informed consent resulted in significantly different response rates among sites and affected sample generalizability.

## Study objectives

The overarching goals of this study are to promote high ethical standards in cluster randomized trials and to promote uniformity in the ethics review of cluster randomized trials through the development of explicit guidelines. Specific objectives are to:

1. Identify challenges arising in the ethical conduct of cluster randomized trials through examination of published trial reports and elicitation of the views and experiences of researchers (cluster randomization trialists);

2. Identify challenges arising in the ethical review of cluster randomized trials through elicitation of the views and experiences of researchers and REBs;

3. Elicit the views of research participants (patients, members of the public, and gatekeepers or cluster level decision-makers) regarding ethical issues in cluster randomized trials;

4. Develop well-grounded guidelines for the ethical conduct and review of cluster randomized trials by conducting an extensive ethical analysis and organizing a consensus process;

5. Disseminate the guidelines to researchers, REBs, journal editors and funders of research.

### Work Leading up to this Study

In preparation for this research study, we conducted interviews with 22 key informants, including ethicists, statisticians, and trialists experienced in cluster randomized trials. The interviews were conducted using a semi-structured interview guide and addressed informants' experiences with the ethics review process and ethical challenges arising in cluster randomized trials. Interviews were conducted by telephone and were audio-taped with the participants' consent. Audio-tapes were transcribed verbatim, verified by the interviewer, and imported into a qualitative software package NVivo 8 (QSR, Inc. Victoria, Australia) to facilitate thematic coding, evaluation and analysis. This necessary preliminary work was carried out to generate an initial framework of ethical issues arising in cluster randomized trials, as seen from a variety of research perspectives. The framework will continue to be updated as the study progresses. Results from the key informant interviews and qualitative analysis will be published elsewhere.

## Methods

**Figure 1:**
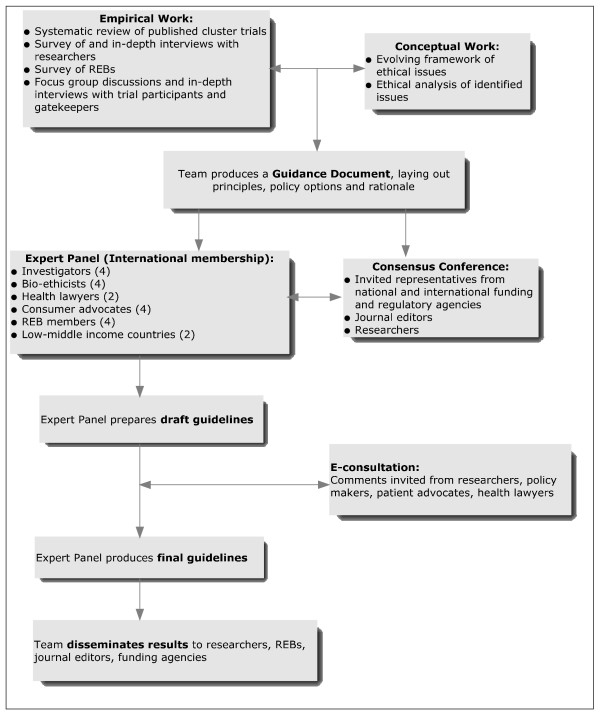
**Outline of the research plan**.

Our study design utilizes a mixed-methods (qualitative and quantitative) approach incorporating both empirical and conceptual work. An outline of the study design is presented in Figure 1.

### Overview

Empirical work will include: a systematic review of a random sample of published cluster randomized trials; a survey of cluster randomization trialists supplemented by in-depth interviews; a survey of REBs; and focus group discussions and in-depth interviews with trial participants and gatekeepers (cluster level decision-makers). The empirical work will inform the concurrent conceptual (ethical) analysis which will lead to a guidance document laying out principles, policy options, and rationale for recommendations. An Expert Panel will be appointed and a consensus conference will be convened. Draft guidelines will be generated by the Panel and an e-consultation phase will be launched. Guidelines will then be revised by the Panel and widely disseminated by the research team.

### Empirical work

#### Systematic Review of Published Cluster Randomized Trials

We will conduct a systematic review of a random sample of 300 cluster randomized trials in health research published between 2000 and 2008. A sampling frame of reports of cluster randomized trials will be identified in Medline by implementing an electronic search strategy (with sensitivity 90.1%) that has been developed and validated by our team [Taljaard M, McGowan J, Grimshaw J et al. 2009. *Electronic search strategies for identifying cluster randomized trials in Medline*. Unpublished Manuscript]. We will select a random sample of 300 eligible trials from the identified reports using computer-generated random numbers. Specific inclusion and exclusion criteria for trials are summarized in Appendix 1.

Using data abstraction forms that have been developed and extensively pilot tested by the research team, we will abstract data on characteristics of the study design, interventions, and outcomes collected; details of informed consent procedures, if any, at individual and cluster level; and details of the ethics review process. Initial samples of 20 trials will be used for reviewer calibration. Thereafter, two reviewers will independently abstract data from each trial report. Agreement between reviewers will be assessed using kappa statistics, with differences resolved by discussion. Frequency distributions will be generated to compare the prevalence of identified ethical issues such as informed consent procedures among study disciplines, types of randomization units, types of interventions, over time, and other descriptors. The sample size of 300 trials is sufficient to give a 95% two-sided confidence interval extending ± 5.7% from an observed proportion of 50%. Results from the systematic review will be used to inform the ethical analysis and to help develop survey questionnaires and interview guides for further empirical work.

#### Survey and In-depth Interviews with Cluster Randomization Trialists

As all ethical issues of interest are unlikely to be fully reported in published articles, the systematic review will be supplemented by a mail survey of corresponding authors of the included trial reports. The survey will consist primarily of closed-ended questions and will elicit trialists' views on ethical issues in cluster randomized trials in general, and gather further information on the published trial included in the systematic review, including consent procedures used at the individual and/or cluster level; ethical challenges arising in the trial; trialists' experiences and satisfaction with the ethics review process; REB queries and concerns related to the submitted trial; perceived impact of the ethics review process on the trial; and uniformity of process and decisions among different REBs involved in the application.

Given the anticipated diversity of geographic locations, academic disciplines, and professions of potential participants, we have selected the mode of delivery to be by mail. The survey will be administered using Dillman's tailored design method [25]. A series of five contacts with trialists is planned as repeated contact is the most effective way to increase response rates. First, a pre-notification letter will be sent by postal mail, explaining the objectives of the survey and identifying the specific trial targeted by the survey. One week later, the questionnaire will be sent by postal mail together with a token financial incentive and a stamped return envelope. A thank-you/reminder postcard will be mailed one week and a replacement questionnaire two weeks later. As a special contact has been shown to improve overall response to mail surveys [25], the remaining nonrespondents will be e-mailed the questionnaire as a printable PDF attachment three weeks later, with instructions for faxing or mailing back the questionnaire. If they wish not to complete the survey, they will be asked the reason(s) for non-participation.

Preliminary analyses using descriptive statistics will be conducted to identify potential non-response bias, by comparing characteristics of respondents and non-respondents using information available in the published reports (e.g., type of randomization unit, discipline, type of intervention). Descriptive statistics will be used to summarize and compare responses, for example, among countries, disciplines, and types of interventions. Results from the survey will be compared with results from the systematic review to assess adequacy of reporting of ethical issues.

A non-random sample of respondents to the mail survey will be identified for in-depth interviews: respondents will be purposefully selected to represent a broad range of views and experiences, using the concept of maximum variation [26]. Interviews will be conducted by telephone using a semi-structured interview guide designed to elicit more in-depth responses than was possible in the mail surveys, using follow-up questions, prompts and probes as needed. In addition to further elaboration of their mail survey responses, a separate section will address new issues arising from the ongoing ethical analysis. We will also request copies of REB application forms and correspondence, which will be imported along with transcripts into NVivo 8™ to facilitate thematic coding, evaluation, and analysis. The purposive sample size will be determined on the basis of theoretical saturation, when new data no longer bring additional insights to the research topic [27]. Experience has shown that a sample size of 12 to 20 is commonly needed when looking for disconfirming evidence or trying to achieve maximum variation [26].

#### Survey of Research Ethics Boards

We will conduct a mail survey of REBs in Canada, the UK, and the USA. A sampling frame of REBs from these countries will be constructed using the websites of the National Council on Ethics in Human Research in Canada [28], the Central Office for Research Ethics Committees (COREC) in the UK [29], and the Office for Human Research Protections (OHRP) in the US [30]. Eligibility criteria include REBs that are (i) currently active, and (ii) review biomedical research – thus, REBs classified as social or behavioural sciences research only will be excluded, as they are unlikely to review cluster randomized trials in health research. We will likely need to include all eligible REBs in Canada and the UK and a probability sample from the USA, for a total sample size of 300. The survey will be administered following Dillman's tailored design method [25] as described above.

We will determine whether the REBs have any experience in reviewing cluster randomized trials, whether they have any training and/or recommendations in place for reviewing cluster trial protocols, whether any ethical challenges with respect to cluster randomized trials have been addressed, and solicit their views and experiences regarding ethical issues in cluster randomized trials. We will also conduct a content analysis of their policy statements, ethics application forms and supporting documents in order to learn the extent and nature of existing guidelines and recommendations relevant to cluster randomized trials. An internet search will be conducted to download the relevant documents online; if not available, REBs will be requested to send copies by mail. The results will be used to inform the ethical analysis and to help develop knowledge translation activities to REBs.

#### Focus Group Discussions and In-depth Interviews with Trial Participants and Gatekeepers

We will conduct focus group discussions and in-depth interviews with trial participants or potential participants in cluster randomized trials. The views and experiences of individuals (e.g., patients, citizens, students), participants at the cluster level (e.g., health professionals, program staff, teachers), and gatekeepers (e.g., community representatives or cluster decision-makers) will be elicited. Semi-structured interview guides and discussion guides will likely be scenario-based and will be developed by the research team upon completion of the systematic review of published trials, which will provide a rich source of information for scenario development. The specific ethical issues to be addressed during the interviews and focus group discussions will be identified in the ethical analysis. Discussions and interviews will be audio-taped, transcribed verbatim and verified by the facilitator/interviewer prior to analysis. Data analysis will be consistent with the methods described by Marshall and Rossman [31] and Crabtree and Miller [26].

### Conceptual Work

#### Ethical Analysis

The ethical analysis will be an intensive process which will take place concurrently with the empirical work. Conceptual work in ethics is not amenable to the degree of *a priori *methodological specification that is expected of empirical research. Reproducibility is an indispensable feature of rigorous science, necessitating the clear statement of hypotheses and experimental methods upfront; rigorous conceptual work in ethics, on the other hand, begins with the articulation of clear and important questions and is realized in the construction of careful moral arguments in peer-reviewed publications and policy reports.

Analysis of the key informant interviews conducted in preparation for this research study has led to identification of an initial framework of ethical questions to be addressed (presented in Appendix 2). This evolving framework will be updated using results from the systematic review, and the trialist and REB surveys. For each ethical issue identified, an in-depth ethical analysis will be prepared. The analysis will begin with a detailed review of relevant foundational documents in research ethics, such as the publications of the US National Commission for the Protection of Human Subjects of Biomedical and Behavioural Research [3,32]. The analysis will also encompass relevant guidelines of different government agencies, ethical codes of professional associations, legal standards, accreditation standards, and reports of (other) advisory committees. For this purpose, we will consult websites such as those of the Canadian Interagency Advisory Panel on Research Ethics [33]; the US President's Advisory Council on Bioethics [34]; the UK Research Ethics Framework project of the Economic and Social Research Council [35], which collect links to human research ethics norms (policies, laws and guidelines); selected organizations involved in the ethics of human research; and other resources in Canada, the UK, Europe and the world. An extensive review of the scholarly literature will document and critically analyze arguments proffered for and against ethical positions. The ethical analysis will seek to synthesize foundational documents, regulations, and arguments in the literature into a coherent position. Where disagreement among the various sources cannot be resolved by critical analysis, the contours of the ethical dispute will be documented. The ethical analysis for each identified ethical issue will result in preparatory papers which will be subjected to in-depth discussion at regular face-to-face meetings of the research team, which represents both ethicist (CW, RS, AB, AM) and trialist perspectives (JG, MPE, AD, MZ, RB). Each preparatory paper will lead to an extensive background document which will be submitted for peer-reviewed publication; documents will also be provided to the members of the guideline writing committee to serve as a foundation for their deliberations.

#### Expert Panel and Consensus Process

An Expert Panel of 20 members will be identified to develop guidelines for the ethical conduct and review of cluster randomized trials. The composition of the Panel will include: 4 investigators (of which 2 will be members of the research team); 4 ethicists (2 research team members, 1 independent member from the UK and 1 from the USA); 2 health lawyers; 4 consumer advocates (2 from Canada and 1 each from the USA and the UK); 4 REB representatives (2 Canadian, 1 USA, 1 UK); and 2 representatives from low-middle income countries. The Panel will be provided with the documents prepared during the ethical analysis and will have a series of preparatory meetings to discuss the documents in advance of a one-day consensus conference, attended by invited representatives from research councils, government and funding agencies, researchers, and journal editors. Following the consensus conference, the Panel will prepare a draft guideline document, including recommendations with rationale and supporting examples. An e-consultation process will be launched to invite comments from the wider research community, policy makers, patient advocates and members of the public. Participants will be invited to make their views known on the website, by e-mail or at an answering service. Based on results from the e-consultation process, the Panel will make revisions and produce final guidelines. The research team will prepare manuscripts and supporting documents for dissemination.

## Discussion

There has been a proliferation of cluster randomized trials in health research in recent years and their use is likely to increase in both primary and secondary care and public health. The importance of improving the quality of patient care is recognized by governments world-wide; new evidence on effective treatments and therapies is being produced on an ongoing basis, but such findings will not impact patient care unless health care professionals adopt them in practice [36]. However, strategies to change health care professional practices and service delivery themselves require rigorous evaluation in real-world settings; cluster randomized trials in which entire medical practices or health care units are randomized, are ideal for this purpose. Likewise, in public health there is a growing concern to improve policies and programs. Cluster randomization is often the ideal or the only feasible approach to evaluate alternative policies and models of care. The basic principles underpinning the ethical conduct of research involving human beings as reflected in international ethics codes have become enshrined in the ethics review of biomedical research; however, they do not have a clear-cut interpretation in cluster randomized trials. This research will address uncertainties and inconsistencies in the conduct and review of cluster randomized trials: we will identify the most important ethical issues arising in cluster randomized trials in a wide variety of different settings, develop a widely accepted set of guidelines which can be used by trialists, REBs and journal editors to ensure that the highest ethical standards are adhered to, and suggest practical ways to improve review of such trials. Ultimately, this should result in improvements in the conduct of cluster randomized trials, accountability, and protection of human subjects in research.

## Competing interests

MT, CW, JB, AB, JCB, SHC, AM, RS: None declared

JG, MPE, RB, MZ and AD have all submitted cluster trial protocols to ethics committees and had difficulty explaining to them the differences between cluster randomized trials and individual patient clinical trials.

## Authors' contributions

MT, CW, JMG, MPE, RB, JCB, RS, MZ and AD contributed to the conception and design of the study. MT and CW led the writing of the manuscript. All authors commented on sequential drafts and approved the final version.

## Appendix 1

### Inclusion and exclusion criteria for the systematic review

#### Inclusion criteria

(i) Random allocation by cluster;

(ii) English language;

(iii) Year of publication 2000 or later;

(iv) Outcomes of interest pertain to individual or population health;

(v) At least some outcomes observed on (or aggregated from) individuals within clusters.

#### Exclusion criteria

(i) Quasi-randomized design;

(ii) Further random or non-random allocation of individuals within clusters;

(iii) Use of standardized patients only;

(iv) Pilot or feasibility studies;

(v) Trial protocols or methods papers;

(vi) Obviously secondary analyses of trials with main results published elsewhere;

(vii) Short communications, conference proceedings, letters to editor;

(viii) Studies randomizing households, or dyads of different individuals (e.g., patient-caregiver, parent-child).

## Appendix 2

### Draft questions to be addressed in the ethical analysis

1. What constitutes a research intervention? Must it be an intervention specifically done to a subject? Or does manipulation of an individual's environment constitute a research intervention (e.g. public advertising or altering a health professional's practice pattern)?

2. Who is the research subject? Individuals who contribute data? Individuals who receive the experimental intervention, including health professionals?

3. Must informed consent be obtained from all research subjects? If all members of a cluster will be affected by the study intervention, must all consent to study participation? If yes, how ought one deal with circumstances in which at least one member of a cluster declines study participation?

4. If informed consent is not required in all cases, under what circumstances is it required? What role ought a waiver of consent play in cluster randomized trials in health?

5. Must informed consent be obtained before randomization? If not, when is post-randomization consent permissible? What must research subjects be told in such circumstances?

6. What is a gatekeeper? How ought the gatekeeper be identified? Are there different criteria for identifying gatekeepers depending on the type of cluster? What is the scope of the gatekeeper's authority? Is the consent of the gatekeeper on behalf of the cluster ever sufficient (that is, consent from members of the cluster is not required)? If so under what circumstances? What criteria should a gatekeeper use when deciding whether or not the cluster should participate in a cluster trial (i.e. some sort of best-interest standard, or other criteria)?

7. How are considerations in #6 impacted if the cluster is a medical practice? Do physicians have a professional obligation to participate in research that may improve practice? May a physician decline to participate in a CRT if participation offers the prospect of therapeutic benefit to her patients?

8. What is the moral basis for the permissibility of random intervention assignment? Can the concept of clinical equipoise apply to cluster randomized trials? If not, what criteria may be used to ensure that clusters and subjects are not disadvantaged by random assignment to one intervention or another?

9. How ought the ethical analysis of benefits and harms be performed a) for subjects that receive the target intervention; b) for subjects who simply contribute data?

10. What are key justice issues with respect to subject selection and cluster selection? How should the benefits and burdens of research participation be distributed between individual subjects and between clusters?
